# New evidence of nematode-endosymbiont bacteria coevolution based on one new and one known dagger nematode species of *Xiphinema americanum*-group (Nematoda, Longidoridae)

**DOI:** 10.1371/journal.pone.0217506

**Published:** 2019-06-26

**Authors:** Mahyar Mobasseri, Matthew C. Hutchinson, Farahnaz Jahanshahi Afshar, Majid Pedram

**Affiliations:** 1 Department of Plant Pathology, Faculty of Agriculture, Tarbiat Modares University, Tehran, Iran; 2 Department of Ecology and Evolutionary Biology, Princeton University, Princeton, New Jersey, United States of America; 3 Iranian Research Institute of Plant Protection, Agricultural Research, Education and Extension Organization (AREEO), Tehran, Iran; Oklahoma State University, UNITED STATES

## Abstract

Three populations of *Xiphinema primum* n. sp. and two populations of *X*. *pachtaicum* were recovered from natural forests and cultural regions of northern Iran. Both species belong to the *X*. *americanum*-group and were characterized by their morphological, morphometric and molecular data. The new species, which was recovered in three locations, belongs to the *X*. *brevicolle*-complex and is characterized by 2124–2981 μm long females with a widely rounded lip region separated from the rest of the body by a depression, 103–125 μm long odontostyle, two equally developed genital branches with endosymbiont bacteria inside the ovary, which are visible under light microscope (LM), vulva located at 51.8–58.0%, the tail is 26–37 μm long with a bluntly rounded end and four juvenile developmental stages. It was morphologically compared with nine similar species *viz*. *X*. *brevicolle*, *X*. *diffusum*, *X*. *incognitum*, *X*. *himalayense*, *X*. *luci*, *X*. *parabrevicolle*, *X*. *paramonovi*, *X*. *parataylori* and *X*. *taylori*. The second species, *X*. *pachtaicum*, was recovered in two geographically distant points close to city of Amol. Molecular phylogenetic studies of the new species were performed using partial sequences of the D2-D3 expansion segments of the large subunit ribosomal RNA gene (LSU rDNA D2-D3), the internal-transcribed spacer rDNA (ITS = ITS1+5.8S+ITS2), and the mitochondrial cytochrome c oxidase I gene (*COI* mtDNA) regions. The Iranian population of *X*. *pachtaicum* was also phylogenetically studied based upon its LSU rDNA D2-D3 sequences. Both species were also inspected for their putative endosymbiont bacteria. *Candidatus* Xiphinematobacter sp. was detected from two examined populations of the new species, whereas the second endosymbiont bacterium, detected from three examined isolates of *X*. *pachtaicum*, was related to the plant and fungal endosymbionts of the family Burkholderiaceae. The phylogenetic analyses of the two endosymbiont bacteria were performed using partial sequences of 16S rDNA. In cophylogenetic analyses, significant levels of cophylogenetic signal were observed using both LSU rDNA D2-D3 and *COI* mtDNA markers of the host nematodes and 16S rDNA marker of the endosymbiont bacteria.

## Introduction

The nematode species of the genus *Xiphinema* Cobb, 1913 [1] are migratory ectoparasites of roots in a wide range of plants. They cause damage through direct feeding on the root cells of host plants and also through the transmission of some pathogenic plant nepoviruses [2]. Species of this genus are traditionally and historically divided into two groups, those of the *americanum*-group, and those of non-*americanum*-group [3–6].

The *X*. *americanum*-group species are generally small nematodes (compared to non-*americanum* species; usually, 1–3 mm long females) that are unified by the morphological similarities in the lip region, a spiral body after relaxation, and in genital tracts characters (for the revised grouping characters, see Lamberti et al. [5]). Historically, Lima [7] and Tarjan [8] noted the species with these morphological similarities as the *X*. *americanum*-complex. Species delimitation has been an issue due to their close morphology and usually overlapping morphometric ranges [9] and thus yielded a “complexity” of their identification, and designation of the *X*. *americanum*-complex. Mean values of morphometric data ranges have recently been proposed for primary clustering of populations [10]. The aforementioned “complex”, is nowadays known as *X*. *americanum*-group, mainly after exploiting high-throughput molecular techniques for species delimitation [11], which are now routinely applied [12–18]. On the other hand, available molecular data is lacking for most species of the group. Lamberti et al. [5, 19] listed 49 species in this group, including some species synonymized by Luc [4]. Since 2004, 10 extra species have been added to that group (Table 1).

**Table 1:** *Xiphinema americanum*-group spp. described after 2004 (in chronological order).

Species	Reference
*X*. *parasimile* Barsi & Lamberti, 2004	Barsi & Lamberti [20]
*X*. *parabrevicolle* Gutiérrez-Gutiérrez, Cantalapiedra-Navarrete, Decraemer, Vovlas, Prior, Palomares-Rius & Castillo, 2012	Gutiérrez-Gutiérrez et al. [14]
*X*. *parapachydermum* Gutiérrez-Gutiérrez, Cantalapiedra-Navarrete, Decraemer, Vovlas, Prior, Palomares-Rius & Castillo, 2012	Gutiérrez-Gutiérrez et al. [14]
*X*. *paratenuicutis* Gutiérrez-Gutiérrez, Cantalapiedra-Navarrete, Decraemer, Vovlas, Prior, Palomares-Rius & Castillo, 2012	Gutiérrez-Gutiérrez et al. [14]
*X*. *plesiopachtaicum* Archidona-Yuste, Navas-Cortés, Cantalapiedra-Navarrete, Palomares-Rius & Castillo, 2016	Archidona-Yuste et al. [18]
*X*. *vallense* Archidona-Yuste, Navas-Cortés, Cantalapiedra-Navarrete, Palomares-Rius & Castillo, 2016	Archidona-Yuste et al. [18]
*X*. *astaregiense* Archidona-Yuste, Navas-Cortés, Cantalapiedra-Navarrete, Palomares-Rius & Castillo, 2016	Archidona-Yuste et al. [18]
*X*. *browni* Lazarova, Peneva & Kumari, 2016	Lazarova et al. [16]
*X*. *penevi* Lazarova, Peneva & Kumari, 2016	Lazarova et al. [16]
*X*. *parataylori* Lazarova, Oliveira, Prior, Peneva & Kumari, 2019	Lazarova et al. [21]

From an agricultural vantage point, some species of the group are regarded as quarantine pests. Indeed, in Europe, the European Economic Union has ratified funding for projects dealing with the identification of virus vector species [5]. Some species are also rated as A1 and A2 quarantine nematodes by the European and Mediterranean Plant Protection Organizations [10, 20]. The species *X*. *pachtaicum* (Tulaganov, 1938 [22]) Kirjanova, 1951 [23] seemingly occurs worldwide, as it is common in in Europe [24–27] and North America [10]. According to Pedram [28], it is common in Iran.

The ability of these nematodes to retain viable plant viruses in absence of host plants adds to their importance as agriculture pests. Indeed, nine species of genus *Xiphinema* have been proven to transmit viruses (genus *Nepovirus*, family Secoviridae [27]), but this could be an underestimate as the capacity of several described *Xiphinema* species to transmit viruses has not examined. By 2000, seven species of the *americanum*-group *Xiphinema*s were known as capable of transmitting plant pathogenic nepoviruses in natural or laboratory conditions [5] and the aforementioned research measures and funding allocation, emphasize the need for reliable species identifications in this group.

Besides the biological ability of *X*. *americanum*-group members to transmit viruses, their relationships with endosymbiont *Candidatus* Xiphinematobacter spp. belonging to subdivision 2 of the Verrucomicrobia is another interesting area of research, already documented and discussed [29–33]. The nematode species in the group have prokaryotic endosymbionts and reproduce by thelytokous parthenogenesis; therefore, endosymbiont bacteria are maternally inherited [31]. The bacteria primarily occupy the gut of juveniles, while their final destination isfemale’s ovaries for transovarial vertical transmission through thelytokous parthenogenesis [30, 32–33]. Long-term association of the nematode with its endosymbiont and species-specific associations are already hypothesized [34]. Recent studies have emphasized identification [16] and cophylogenetic analyses of these endosymbiont bacteria with their host nematodes [10, 34], to corroborate the long-term association and significant cophylogeny between nematode host and endosymbiont. In one of the aforementioned studies [34], a new endosymbiont, belonging to the plant and fungal endosymbionts of the family Burkholderiaceae Garrity, Bell & Lilburn, 2005 [35] was discovered. The cophylogenetic analyses of this group and the host nematodes also demonstrated significant association between phylogenies including species-specific associations (for a few exceptions in species specificity of the endosymbionts, see [34]). In a more recent study, Howe et al. [36] also showed the congruence between the host and endosymbiont phylogeny using genetic markers of varying evolutionary rates.

Several members of the *Xiphinema americanum*-group have a global distribution [5, 6, 11, 14]. Currently nine species of this group have been reported from Iran [28]. During our surveys in two Mazandaran and Tehran provinces conducted in 2015–2017, which followed our previous studies on Longidoridae Thorne, 1935 [37] in the country (the history within [38, 39]), several populations of *X*. *americanum*-group were recovered. Primary morphological studies revealed three of them could be conspecific and do not fit known species of the group thus representing a new species, described herein as *Xiphinema primum* n. sp. The other populations belonged to *X*. *pachtaicum*. Study with light microscopes revealed endosymbiont bacteria occupying ovaries of several females of both species. Accordingly, the present study aims to (*i*) describe *X*. *primum* n. sp. and characterize the recovered populations of *X*. *pachtaicum* using morphological and molecular data, (*ii*) characterize the endosymbiont bacteria of the two nematode species, and (*iii*) perform cophylogenetic analyses using selected LSU rDNA D2-D3 and *COI* mtDNA sequences of host nematodes and 16S rDNA sequences of their endosymbiont bacteria.

## Material and methods

### Ethics statement

Specific permissions were not needed for collecting the nematodes studied. All soil samples were collected from public regions and natural forests, that were not under protection and no endangered animal species were under protection in those regions. The garden samples were collected after taking the permission from the landowner. There was no need for an animal research ethics committee to approve this research.

### Nematode materials

The *X*. *americanum*-group species/populations studied in the present research were recovered from soil samples collected from cultivated fields and natural forests in two Mazandaran and Tehran provinces using the flotation method [40] and direct extraction of the nematodes using a series of 20, 60 and 270 mesh sieves (USA standard mesh numbers), equal to 850, 250, 53 μm openings size, respectively.

### Morphological observations

The hand-picked specimens were killed with 4% formalin solution, transferred to anhydrous glycerin according to De Grisse [41], and mounted on permanent slides. Photographs were taken using an Olympus DP72 digital camera attached to an Olympus BX51 microscope powered with differential interference contrast (DIC). Drawings of the new species were made using a drawing tube attached to the microscope and were redrawn using CorelDRAW software version 16.

### Molecular profiles

For DNA extraction, each nematode was washed with distilled water, surface sterilized using sodium hypochlorite (0.5% v/v) for 5 min, or 5% (v/v) solution of commercial Dettol for 1 min., followed by washing in distilled water three times, transferred to an Eppendorf tube containing 1 μl proteinase K (CinnaGen, Tehran) (10 mg/ml) and 49 μl of extraction buffer (worm lysis buffer [42]; containing: 50 mM KCL, 10 mM Tris-Cl pH 8.3, 2.5 mM MgCl2, 0.45% NP40, and 0.45% Tween 20), frozen at −80°C (20 min), incubated at 65°C (2 h), and consequently treated at 95°C (10 min) for inaction of the proteinase K enzyme. DNA samples were stored at −20°C until their use as PCR templates. PCR was carried out in a total volume of 30 μl [19.2 μl distilled water, 3 μl 10× PCR buffer, 0.6 μl 10 mM dNTP mixture, 1.2 μl 50 mM MgCl2, 1.2 μl of each primer (10 pmol/μl), 0.6 μl of *Taq* DNA polymerase (5 unit/μl, CinnaGen, Tehran, Iran) and 3 μl of DNA template]. The thermal cycling program for amplifying three nematode genomic fragments (SSU, LSU D2-D3 and ITS rDNA) was as follows: denaturation at 95°C for 4 min, followed by 35 cycles of denaturation at 94°C for 30 s, annealing at 52°C for 40 s, and extension at 72°C for 80 s. A final extension was performed at 72°C for 10 min. The *COI* mtDNA was amplified according to aforementioned PCR program, except the annealing temperature was set to 46°C. The PCR mixture rations and the thermal cycling program for amplifying bacterial 16S rDNA was as already described for nematode genomic fragments amplifications.

The information of the primers used for amplification of aforementioned fragments are given in Table 2.

**Table 2:** Primers used to amplify and sequence nematode and bacterial ribosomal, and nematode mitochondrial DNA.

Genomic or mitochondrial fragment	Primer name	Direction	Primer sequence 5′ - 3′	Reference
SSU rDNA	1096 F	Forward	GGTAATTCTGGAGCTAATAC	Holterman et al. [43]
SSU rDNA	1912R	Reverse	TTTACGGTCAGAACTAGGG	Holterman et al. [43]
SSU rDNA	1813F	Forward	CTGCGTGAGAGGTGAAAT	Holterman et al. [43]
SSU rDNA	2646R	Reverse	GCTACCTTGTTACGACTTTT	Holterman et al. [43]
D2-D3 rDNA	D2A	Forward	ACAAGTACCGTGAGGGAAAGT	Nunn [44]
D2-D3 rDNA	D3B	Reverse	TGCGAAGGAACCAGCTACTA	Nunn [44]
LSU rDNA	KK28S-1	Forward	AAGGATTCCCTTAGTAACGGCGAGTG	Kiontke et al. [45]
LSU rDNA	KK28S-4	Reverse	GCGGTATTTGCTACTACCAYYAMGATCTGC	Kiontke et al. [45]
ITS rDNA	rDNA1	Forward	TTGATTACGTCCCTGCCCTTT	Subbotin et al. [46]
ITS rDNA	rDNA1.58S	Reverse	ACGAGCCGAGTGATCCACCG	Subbotin et al. [46]
ITS rDNA	rDNA2	Reverse	TTTCACTCGCCGTTACTAAGG	Subbotin et al. [46]
*COI* mtDNA	COIF	Forward	GATTTTTTGGKCATCCWGARG	He et al. [47]
*COI* mtDNA	COIR	Reverse	CWACATAATAAGTATCATG	He et al. [47]
16S rDNA	360F	Forward	AGCAACGCCGCGTGGAGGATGAA	Lazarova et al. [48]
16S rDNA	920R	Reverse	ATCGAATTAAGCCACATACTCCA	Lazarova et al. [48]
16S rDNA	27F	Forward	AGAGTTTGATCCTGGCTCAG	Brosius et al. [49]
16S rDNA	1541R	Reverse	AAGGAGGTGATCCAGCCGCA	Brosius et al. [49]

The PCR products were sequenced in both directions using the same primers with an ABI 3730XL sequencer (Applied Biosystems) at Macrogen (Seoul, South Korea). Newly obtained sequences of the studied nematode species and their endosymbiont bacteria were deposited into the GenBank database under the accession numbers given in Table 3.

**Table 3:** The species and isolate names, newly generated accession numbers and the sequenced regions.

Sequenced specimens			Genomic or mitochondrial regions and the accession numbers				Bacterial endosymbiont and the accession number
Species	Isolate code	Sequenced specimen	SSU rDNA	LSU rDNA	*COI* mtDNA	ITS rDNA	16S rDNA of the associated endosymbiont
*X*. *pachtaicum*	Dtiz1	1 female	-	MF372944	-	-	Burkholderiaceae bacterium, MF372949
*X*. *pachtaicum*	Dtiz2	1 female	-	MF372945	-	-	Burkholderiaceae bacterium, MF372950
*X*. *pachtaicum*	Dtiz3	1 female	-	MF372946	-	-	Burkholderiaceae bacterium, MF372948
*X*. *pachtaicum*	Dtiz4	1 female	MF372940	-	-	-	-
*X*. *primum* n. sp.	D1-Ta	1 female	-	MF372947	MK202795	MF372951	*Candidatus* Xiphinematobacter sp., MF372956
*X*. *primum* n. sp.	D2	1 female	-	-	-	MF372952	-
*X*. *primum* n. sp.	Dkond1	1 female	-	MF372942	-	MF372953	*Candidatus* Xiphinematobacter sp., MF372955
*X*. *primum* n. sp.	Dkond2	1 female	-	MF372943	-	MF372954	-
*X*. *primum* n. sp.	Dbana	1 female	-	MF372941	-	-	-
*X*. *primum* n. sp.	Ka	1 female	-	-	MK202796	-	-

### Phylogenetic analyses

The newly obtained SSU, LSU D2-D3, ITS, *COI* mtDNA and 16S rDNA sequences were compared with those of other nematodes and bacteria sequences available in GenBank database using the BLAST homology search program.

The DNA sequences for reconstructing the phylogenetic trees were selected according to the recent studies [10, 21, 34, 48]. The selected sequences were updated after BLAST search.

The LSU rDNA D2-D3 and *COI* mt DNA sequences (94 and 75 sequences respectively, including outgroup sequences) were aligned using the MUSCLE algorithm implemented in MEGA 6 [50]; and the resultant alignment was manually edited.

The nematode ITS sequences (53 sequences including outgroup sequences) and the bacterial 16S rDNA (42 sequences for *Candidatus* Xiphinamtobacter spp. and 25 sequences for Burkholderiaceae spp. phylogenies respectively, including outgroup sequences) were aligned using the Q-INS-i algorithm of online version of MAFFT version 7 (http://mafft.cbrc.jp/alignment/server/) [51]. The Gblocks program (version 0.91b) with the three less stringent parameters, a server tool at the Castresana Lab (http://molevol.cmima.csic.es/castresana/ Gblocks_server.html), was used for post-editing of this alignment to eliminate poorly aligned regions or divergent positions.

The most appropriate model of nucleotide substitution was selected using the Akaike information criterion (AIC) in MrModeltest 2 [52]. The general time-reversible model, including a gamma distribution for rates across sites and a proportion of invariant sites (GTR+G+I) was selected for all phylogenetic analyses in this study. Bayesian inference (BI) was performed using MrBayes v3.1.2 [53], running the chains for 5 × 10^6^ generations (for all datasets). After discarding burn-in samples, the remaining samples were retained for further analyses. The Markov-chain Monte Carlo method within a Bayesian framework was used to estimate the posterior probabilities of the phylogenetic trees [54], using the 50% majority rule. Adequacy of the posterior sample size was evaluated using autocorrelation statistics as implemented in TRACER v.1.5 [55]. A maximum likelihood (ML) tree was reconstructed with RaxmlGUI 1.1 [56] software and the same nucleotide substitution model as that used for BI including 1000 bootstrap (BS) pseudoreplicates. For the LSU rDNA dataset, the species *Tylencholaimus teres* Thorne, 1939 [57] and *Ecumenicus monohystera* De Man, 1880 [58], (accession number EF207243, AY593013 respectively), for the ITS rDNA phylogeny, the species *Heterodorus veletensis* Guerrero, Leibanas & Pena-Sañtiago, 2007 [59] (accession number EU477380) and for *COI* phylogeny, *Xiphinema diversicaudatum* (Micoletzky, 1922 [60]) Thorne, 1939 [57] (KF292292) and *X*. *index* Thorne & Allen, 1950 [61] (HM921380) (according to Orlando et al. [10]) were used as outgroup taxa. For the bacterial 16S rDNA phylogeny of *Candidatus* Xiphinematobacter spp., two species *Prosthecobacter fusiformis* Staley, Bont & Jonge, 1976 [62] (U60015) and *Verrucomicrobium spinosum* Schlesner, 1987 [63] (X90515), and for the Burkholderiaceae 16S rDNA phylogeny, the species *Candidatus* Glomeribacter gigasporarum Bianciotto, Lumini, Bonfante & Vandamme, 2003 [64] (AJ251634) were used as outgroup species in accordance with previous studies [10, 34].

The resultant phylogenetic trees were visualized using Dendroscope V.3.2.8 [65] and re-drawn in CorelDRAW software version 16.

### Co-phylogenetic analyses

In order to understand how the new species identified in our study, and its endosymbiont, fit into the broader evolutionary picture of the *Xiphinema americanum*-group, we undertook a cophylogenetic analysis of the nematodes and their endosymbionts. Our analysis examined cophylogenetic signal between the endosymbiont 16S phylogenies and both nematode phylogenies (one based on the LSU sequences, the other based on *COI* sequences). Genetic data was available for nine pairs of host and endosymbiont for the LSU region and seven pairs of species for the COI region. Sequences for the host nematode species were selected when sequences for their endosymbiont bacteria were also available. Cophylogenetic signal in the associations of host and endosymbiont can allude to a parallel evolutionary history between the two as is expected from results elsewhere in similar systems [66–68].

We used the cophylogenetic tool, PACo [69, 70], to perform our analyses. PACo uses a Procrustes approach to superimpose one phylogeny on the other based on the observed host-endosymbiont associations and estimates cophylogenetic signal as the sum of squared residuals (*ss*) from this superimposition [69]. Observed cophylogenetic signal is considered significant if the observed test statistic is significantly smaller than the test statistic observed when the matrix of host-endosymbiont associations is randomized and the analysis re-run [70]. In our analysis, we performed PACo analysis with the asymmetric statistic which assesses the goodness-of-fit of the endosymbiont phylogeny onto the host phylogeny (since we would expect host evolution to drive endosymbiont evolution). We compared our observed cophylogenetic signal to the same from 10,000 randomizations of the association matrix and considered cophylogenetic signal significant if the observed signal was smaller than the random expectation at α = 0.05. We also examined the results from the perspective of individual host-endosymbiont associations to examine how cophylogenetic signal of our newly identified species compared to that between the other host-endosymbiont pairs.

### Nomenclatural acts

The electronic vision of this paper meets the requirements of the amended international code of zoological nomenclature (ICZN), and hence the new name contained herein is available under that code from the electronic vision of this paper. This published work and the contained nomenclatural acts have been registered in the online registration system for the ICZN in ZooBank. The ZooBank LSID (life science identifiers) for this publication is: urn:lsid:zoobank.org:pub:96D34294-16C8-45C2-BEFD-5A0A5C5AA148. The related LSID information can be viewed through any standard web browser by appending the LSID to the prefix "http://www.zoobank.org/References/". The electronic edition of this work was published in a journal with an ISSN, and has been archived and is available from the following digital repositories: PubMed Central, LOCKSS.

## Results

### *Xiphinema primum* Mobasseri, Hutchinson, Jahanshahi Afshar & Pedram n. sp. urn:lsid:zoobank.org:act:6CC653DD-05BE-4F9A-A3BB-990E73B0D2BF

(Figs 1, 2 and 3)

**Fig 1:**
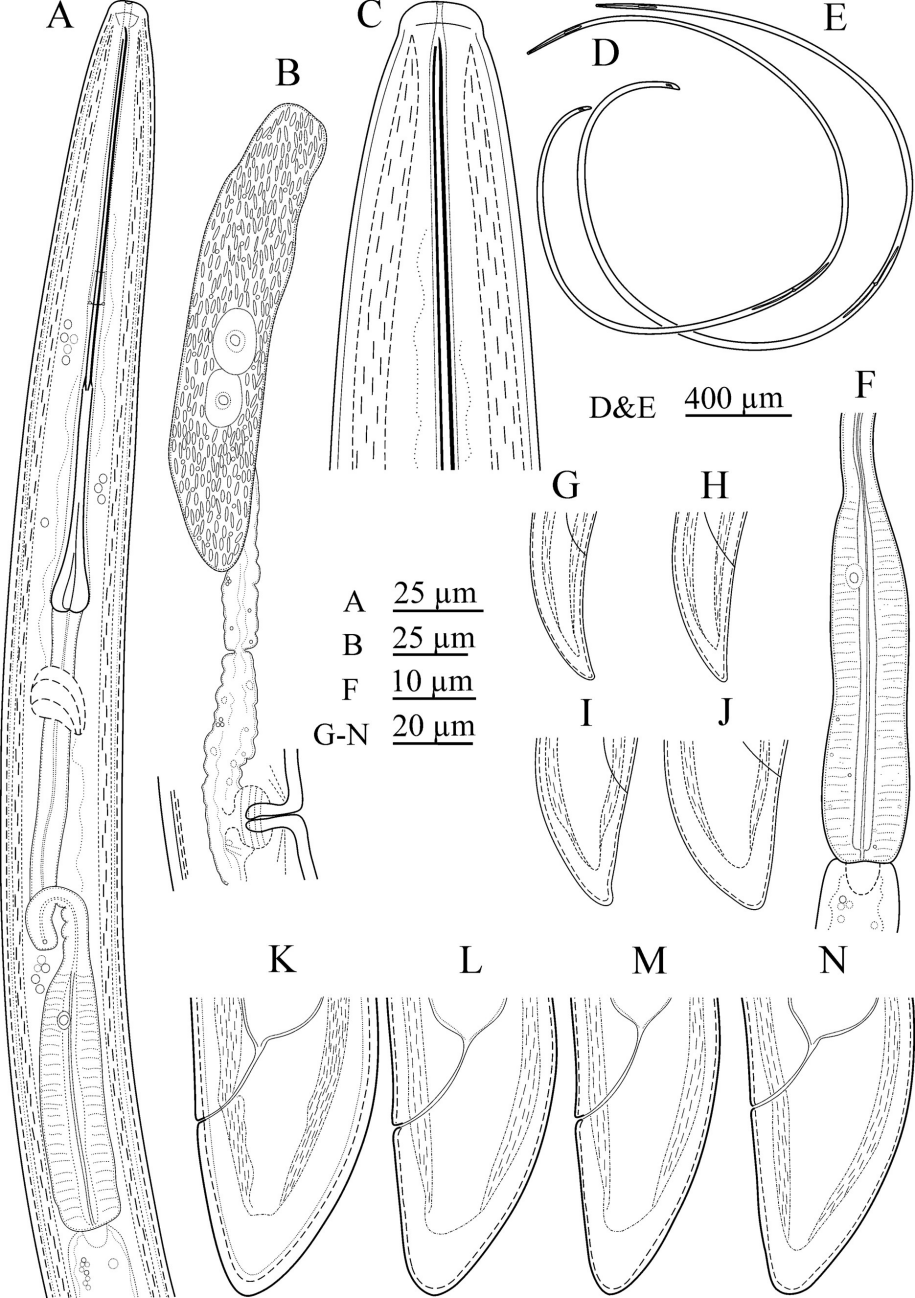
Line drawings of *Xiphinema primum* n. sp. (Amol population). (A) Pharynx. (B) Part of female reproductive system, showing endosymbiont bacteria inside the ovary. (C) Anterior region. (D and E) Entire female. (F) Pharyngeal bulb. (G-J) Tail of juvenile developmental stages from J1-J4, respectively. (K-N) Tail of female.

**Fig 2:**
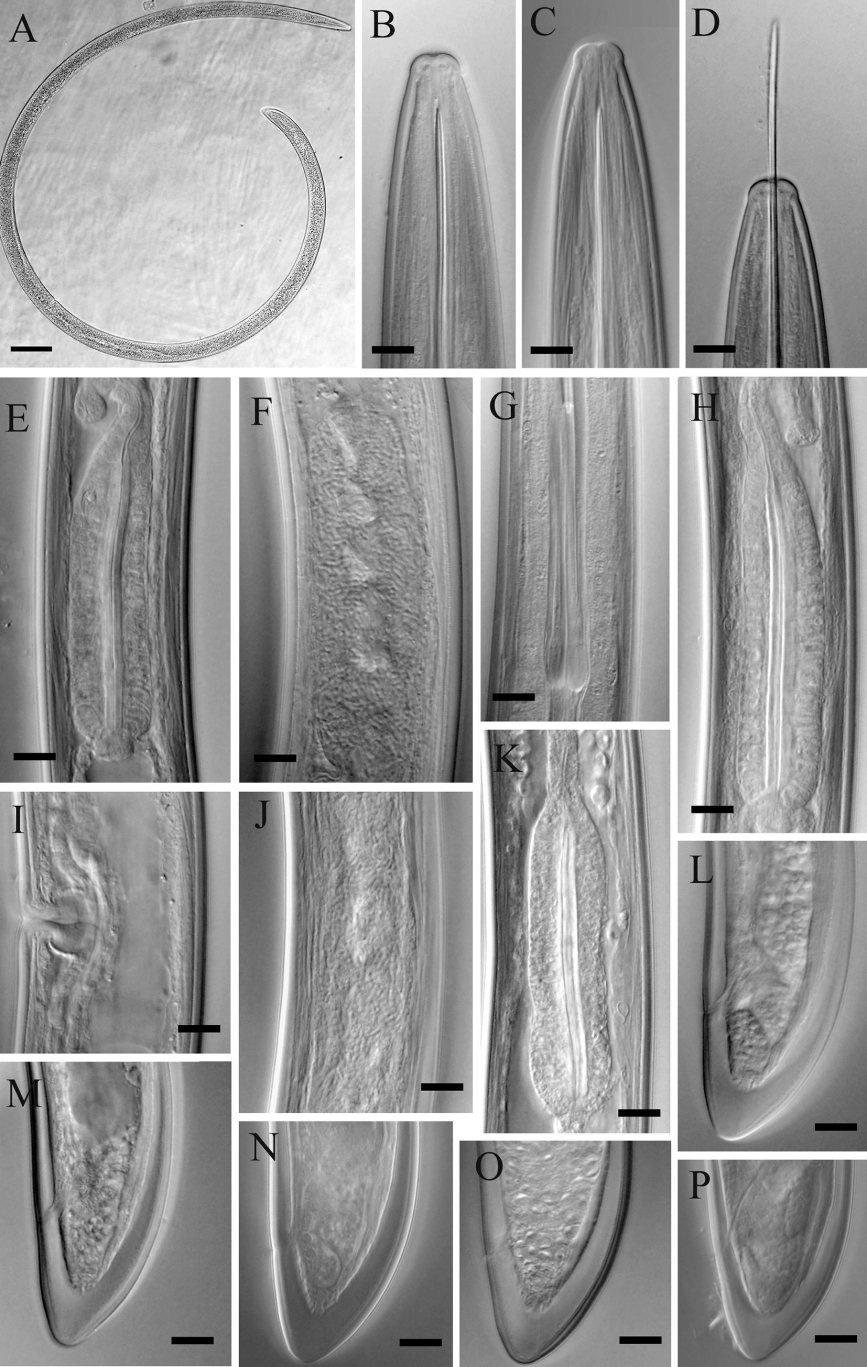
Light microphotographs of *Xiphinema primum* n. sp. (Amol population). (A) Entire female. (B-D) Anterior end. (E, H and K) Pharyngeal bulb. (F and J) Ovary and endosymbiont bacteria. (G) Odontophore. (I) Vulval region. (L-P) Tail of female. (All scale bars = 10 μm, except A = 100 μm).

**Fig 3:**
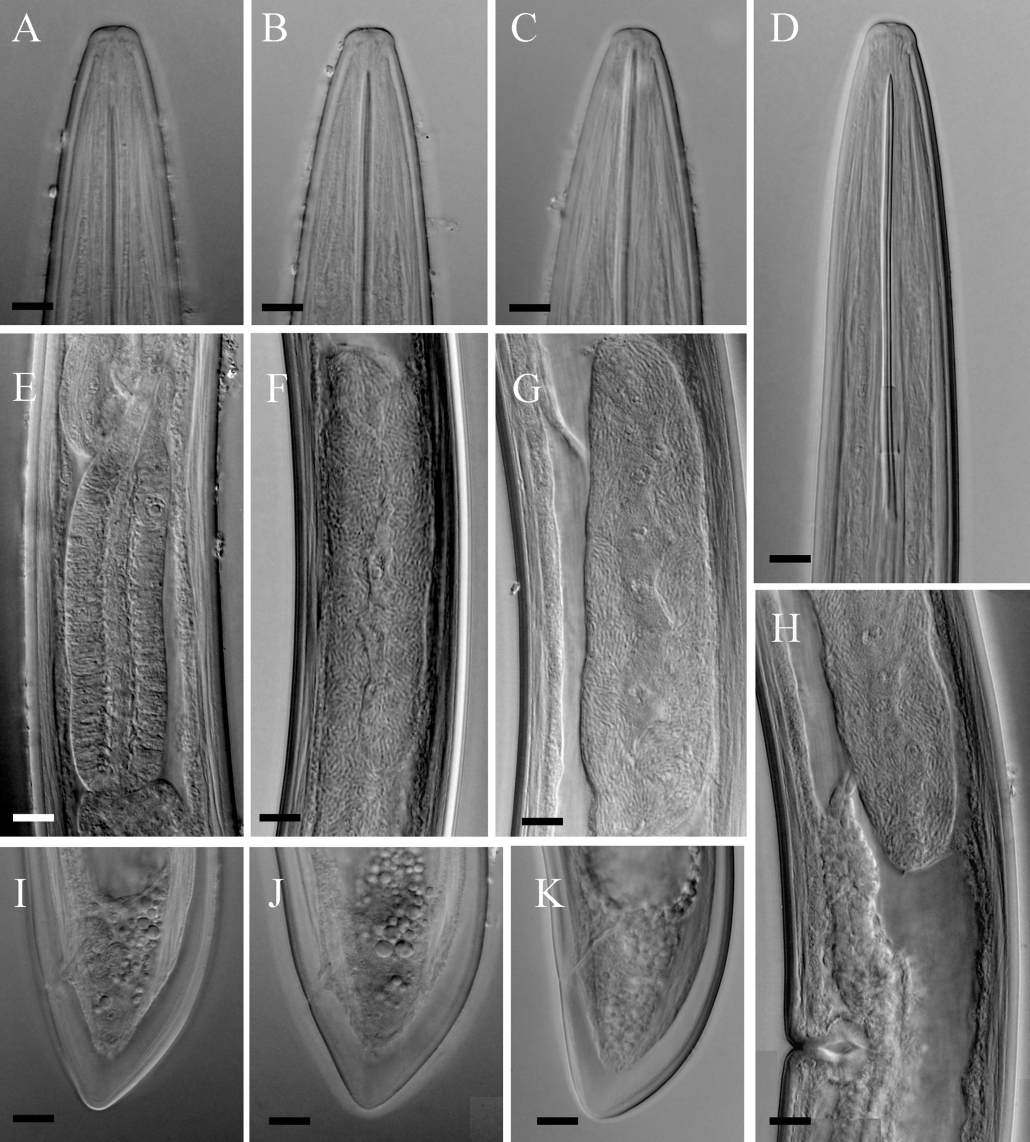
**Light microphotographs of *Xiphinema primum* n. sp. (A-C, E, G, H-J: Tehran population, D, F, K: Ramsar population, paratype female).** (A-D) Anterior region. (E) Pharyngeal bulb. (F and G) Ovary and endosymbiont bacteria. (H) Part of female reproductive system, showing less developed uterus and oviduct. (I-K) Tail of female. (All scale bars = 10 μm).

#### Description

**Female.** Body cylindrical, very gradually narrowing towards both extremities, assuming a loose spiral after fixation (Fig 1D and 1E). Cuticle two layered, smooth under light microscopy, fine transverse striae visible on outer layer mostly in tail. The hyaline part of tail 11–15 μm thick, occupying about 1/3 of tail length. Lateral cord 12–28 μm wide, occupying about 1/4 of corresponding body diam. Lip region widely rounded, separated from the rest body by a shallow depression (Fig 1C and Fig 2B–2D and Fig 3A–3D). Amphidial fovea cup-shaped; aperture 12–13 μm wide, located slightly anterior to lip region–body junction region. Odontostyle ca. 1.7 times longer than odontophore, the latter with well-developed flanges. Guiding ring double and guiding sheath 5–10 μm long depending on the degree of protraction/retraction of the stylet. Pharyngeal bulb 78–85 μm long and 19.5–26.0 μm wide. The larger dorsal gland nucleus (DN) located at 15.3–24.7% of pharyngeal bulb length, the two smaller ventrosublateral nuclei (S1N) located at about the same level, at 50.2–60.3% of terminal bulb length. Cardia 9–19 × 5–13 μm in size. Intestine simple, prerectum often not well seen, and rectum 0.6–0.9 times the anal body diameter. Reproductive system didelphic-amphidelphic, the branches about equal in size, the endosymbiont bacteria within the ovaries visible in most examined individuals (Fig 1B and Fig 2F and 2J and Fig 3F–3H), uterus short *ca* 50 μm long, and the sphincter not clearly seen. Vagina perpendicular to body axis, about 1/2 to 1/3 of corresponding body diameter, the *pars distalis vaginae* 15–20 μm long, the *pars proximalis vaginae* about 1.5 times wider than tall. Tail short, conical, dorsally more convex, with bluntly rounded end (Fig 1K–1N and Fig 2M–2P and Fig 3I–3K). Measurements are listed in Table 4.

**Table 4:** Morphometric data for females of *Xiphinema primum* n. sp. from Iran. All measurements in μm and in the format: mean ± s.d. (range).

Origin	Ramsar (type) population		Tehran population	Amol population	Total range
Characters	Holotype Female	Paratype Females	Female	Female	Female
n	1	6	7	10	24
L	2304	2533.0 ± 312.2	2524.0 ± 179.4	2453 ± 135	2497.7 ± 202.0
		(2124–2981)	(2284–2773)	(2185–2594)	(2124–2981)
a	51.2	44.7 ± 2.0	43.4 ± 3.0	54.1 ± 4.3	48 ± 6
		(42–48)	(39.2–46.9)	(45.5–61.7)	(39.2–61.7)
b	6.5	6.8 ± 0.9	6.4 ± 0.5	6.8 ± 0.3	6.7 ± 0.6
		(5.9–8.2)	(5.7–7.0)	(6.0–7.1)	(5.7–8.2)
c	79.4	76.1 ± 5.4	82.4 ± 7.3	82.9 ± 8.5	81.0 ± 7.7
		(70–83)	(73.7–95.6)	(66.2–95.1)	(66.2–95.6)
c'	1	0.9 ± 0.0	0.8 ± 0.1	0.9 ± 0.1	0.9 ± 0.1
		(0.9–0.9)	(0.8–0.9)	(0.8–1.0)	(0.8–1.0)
V	53.8	53.4 ± 1.3	56.6±1.0	54.2±1.0	54.7 ± 1.7
		(51.8–54.8)	(55.1–58.0)	(51.9–55.3)	(51.8–58.0)
Lip region height	5	5.0 ± 0.9	4.9 ± 0.7	5.1 ± 0.6	5.0 ± 0.7
		(4–6)	(4–6)	(4–6)	(4–6)
Lip region width	12	13.2 ± 1.0	13.0 ± 0.6	11.7 ± 0.7	12.5 ± 1.0
		(12–14)	(12–14)	(11–13)	(11–14)
Odontostyle length	103	115.8 ± 4.7	117.0 ± 4.4	109.0 ± 2.5	113.5 ± 5.3
		(112–125)	(111–122)	(103–112)	(103–125)
Odontophore length	67	65.5 ± 3.6	71.4 ± 2.4	67.8 ± 3.4	68.3 ± 3.8
		(60–70)	(67–74)	(62–74)	(60–74)
Total stylet	170	181.3 ± 7.4	188.4 ± 4.0	176.7 ± 4.6	181.7 ± 7.2
		(173–195)	(184–194)	(170–184)	(170–195)
Anterior end to guiding ring	94	106.7 ± 4.3	100.6 ± 5.7	94.9 ± 2.8	100.0 ± 6.5
		(102–112)	(93–110)	(91–98)	(91–112)
Pharynx	353	371 ± 12	394 ± 18	363 ± 25	375 ± 23.7
		(363–390)	(375–425)	(309–403)	(309–425)
Anterior genital branch	225	253.3 ± 29.3	243.3 ± 6.0	212 ± 37	231.6 ± 33.5
		(213–275)	(223–272)	(154–260)	(154–275)
Posterior genital branch	207	226.4 ± 39.5	228 ± 23	196.8 ± 22.5	214.4 ± 33.5
		(183–265)	(200–258)	(155–221)	(155–265)
Lip region-vulva distance	1239	1350.7 ± 141.8	1428.0 ± 93.6	1329.3 ± 82.5	1366.8 ± 108.8
		(1151–1543)	(1303–1550)	(1187–1420)	(1151–1550)
Body width at mid-body	45	56.7 ± 5.8	58.4 ± 6.4	45.4 ± 2.4	52.5 ± 7.7
		(47–63)	(50–65)	(41–48)	(41–65)
- at anus	30	36.7 ± 3.8	37.6 ± 3.6	32.2 ± 2.8	35 ± 4
		(33–42)	(32–42)	(30–39)	(30–42)
- at base of pharynx	37	47.7 ± 4.0	52.3 ± 4.7	40 ± 4.0	46.0 ± 6.7
		(40–51)	(44–58)	(36–49)	(36–58)
Tail	29	33.3 ± 3.8	30.7 ± 1.5	29.8 ± 2.2	31 ± 3
		(30–37)	(28–32)	(26–33)	(26–37)

**Male.** Not found.

**Fig 4:**
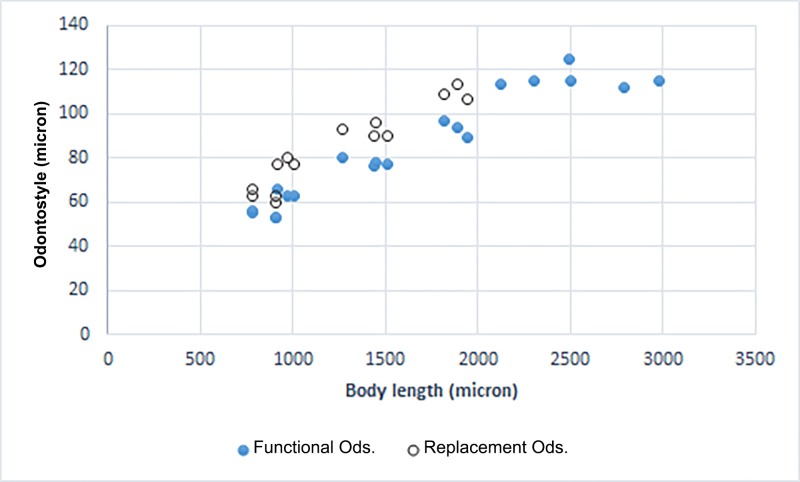
Graph of correlation of functional and replacement odontostyle to body length in juveniles and females of *X*. *primum* n. sp. of the type population with the code D. The replacement odontostyle in each juvenile stage is equal to the functional odontostyle in the next stage, and in J4, is equal to the functional odontostyle in females. (Ods. = odontostyle).

**Juveniles.** Recovered in one of the three populations of the new species (the type population with code D, from Ramsar). All four juvenile developmental stages were identified and separated from each other according to Robbins et al. [71]. The correlation between body length of juveniles and females, replacement odontostyle of juveniles and functional odontostyle of females and juveniles is given in Fig 4. The first juvenile (J1) was characterized by the replacement odontostyle tip close to base of functional odontostyle, located at the level of the odontophore, and three other stages had replacement odontostyle posterior to odontophore flanges in resting position of the spear. All stages had dorsally convex conical tails, with a bluntly rounded terminus, however tail in J1and J2 was narrower (Fig 1G–1J). Measurements are listed in Table 5.

**Table 5:** Morphometric data of paratype juveniles of *Xiphinema primum* n. sp. from Iran. All measurements in μm and in the format: mean ± s.d. (range).

	J1	J2	J3	j4
n	4	3	4	3
L	846.3 ± 73.5	967 ± 43	1418 ± 102	1887 ± 61
	(782.5–9125.5)	(921–1006)	(1272–1511)	(1822–1944)
a	37.4 ± 2.7	40 ± 4	46.5 ± 2.8	45.2 ± 5.4
	(34.8–41.2)	(35.4–42.3)	(44.0–50.5)	(41–51)
b	3.9 ± 0.6	3.7 ± 0.2	4.6 ± 0.4	5.3 ± 0.3
	(3.3–4.5)	(3.5–4.0)	(4–5)	(5.1–5.5)
c	23.4 ± 2.1	29.7 ± 3.3	43 ± 5	57 ± 4
	(21–26)	(28.0–33.5)	(40–49)	(54–62)
c'	2.4 ± 0.2	1.8 ± 0.1	1.5 ± 2.0	1.1 ± 0.1
	(2.2–2.6)	(1.7–1.9)	(1.3–1.6)	(1.0–1.2)
Lip region width	8.8 ± 0.3	9.5 ± 0.0	10.0 ± 0.4	11.3 ± 0.3
	(8.5–9.0)	(9.5–9.5)	(9.5–10.5)	(11.0–11.5)
Odontostyle length	54.3 ± 1.5	64.0 ± 1.7	78.0 ± 1.7	93.3 ± 4.0
	(53–56)	(63–66)	(76–80)	(89–97)
Odontophore length	36.5 ± 1.3	41.3 ± 1.2	48.0 ± 0.8	58.3 ± 2.5
	(35–38)	(40–42)	(47–49)	(56–61)
Stylet total length	91 ± 0 .5	105 ± 0.6	126 ± 1	151.5 ± 1.5
	(90–91)	(105–106)	(125–127)	(150–153)
Replacement odontostyle	63.0 ± 2.4	78.0 ± 1.7	92.3 ± 3.0	109.0 ± 3.5
	(60–66)	(77–80)	(90–96)	(106–113)
Anterior end to guiding ring	42.5 ± 1.8	55 ± 1	68.0 ± 2.3	80.5 ± 0.7
	(41–45)	(54–56)	(66–70)	(80–81)
Pharynx length	221 ± 15	260.0 ± 5.5	305.0 ± 7.3	350 ± 7
	(204–238)	(254–264)	(296–312)	(345–355)
Body width at mid-body	23.0 ± 2.7	24.3 ± 1.5	30.5 ± 1.3	42.2 ± 5.3
	(19–25)	(23–26)	(29–32)	(37.0–47.5)
at anus	15.5 ± 1.0	18 ± 1	22.7 ± 2.3	30.7 ± 1.2
	(14–16)	(17–19)	(20–24)	(30–32)
Tail	36.3 ± 1.3	32.7 ± 2.5	33.0 ± 2.5	33.2 ± 1.8
	(35.0–37.5)	(30–35)	(31–36)	(31.5–35.0)

**Type host and locality**

Rhizospheric soils of an unidentified forest tree in Ramsar, Mazandaran province (the population code D) (GPS coordinates: 36°55′57.22″N, 50°36′47.00″E).

**Other localities**

Two other populations of the new species were recovered from the rhizospheric soil of *Fagus* sp. collected from Amol, Mazandaran province (the population code Dkond), GPS coordinates: 36°24′7.89″N, 52°18′13.054″E; and rhizospheric soil of unidentified trees in village of Āhār, Shemiranat county, Tehran province (the population code Dbana), GPS coordinates: 35°56′13.518″N, 51°27′ 6.615″E.

**Type materials**

Holotype, six paratype females and 14 juvenile paratypes (J1-J4) were deposited in the Nematode Collection at the Faculty of Agriculture, Tarbiat Modares University, Tehran, Iran. Seven females of Tehran population (the Dbana population) were deposited in the USDA Nematode Collection, Beltsville, MD, USA and 10 females of Amol population (the Dkond population) were deposited in Ghent University Museum, Zoology Collections, Ghent, Belgium. Specific sequences are deposited in GenBank with accession numbers given in Table 3.

**Etymology**

The specific epithet shows this is the first species of *X*. *americanum*-group described from Iran.

**Diagnosis and relationships**

*Xiphinema primum* n. sp. belongs to the *brevicolle*-complex of the *Xiphinema americanum*-group, mainly characterized by the lip region being offset from the rest of the body by a slight depression and conical tail, with a bluntly rounded tip. It is further characterized by 2124–2981 μm long females with a widely rounded lip region, separated from the rest of the body by a shallow depression, a 103–125 μm long odontostyle, two equally developed genital branches with visible endosymbiont bacteria under light microscopy, vulva located at 51.8–58.0%, 26–37 μm long conical tail with a more convex dorsal side, bluntly rounded tip and four juvenile developmental stages. The alpha-numeric identification codes of the new species according to Lamberti et al. [19] are: A56, B23, C1, D23, E3, F-, G1, H2, I123.

By having a widely rounded lip region, and conical tail with bluntly rounded tip, the new species comes close to nine species of the *brevicolle*-complex *sensu* Orlando et al. [10] and Palomares-Rius et al. [34] *viz*. *X*. *brevicolle* Lordello & Da Costa, 1961 [72], *X*. *diffusum* Lamberti & Bleve-Zacheo, 1979 [9], *X*. *incognitum* Lamberti & Bleve-Zacheo, 1979 [9], *X*. *himalayense* Ahmad, Lamberti, Rawat, Agostinelli & Srivastava, 1998 [73], *X*. *luci* Lamberti & Bleve-Zacheo, 1979 [9], *X*. *parabrevicolle* Gutiérrez-Gutiérrez, Navarrete, Decraemer, Vovlas, Prior, Palomares-Rius & Castillo, 2012 [14], *X*. *paramonovi* Romanenko, 1981 [74], *X*. *parataylori* Lazarova, Oliveira, Prior, Peneva & Kumari, 2019 [21] and *X*. *taylori* Lamberti, Ciancio, Agostinelli & Coiro, 1991 [75]. The comparisons with aforementioned species are as follows:

Differentiated from *X*. *brevicolle*, besides distant placement in LSU, ITS and *COI* mtDNA trees, by a longer body (2497.7 (2124–2981) *vs* (1800–2200) μm), posteriorly located guiding ring (100 (91–112) *vs* 72.3–92.3 μm) from anterior end and slightly longer tail (31 (26–37) *vs* 23.0–31.2) μm) (data of *X*. *brevicolle* based on topotypes given by Lamberti et al. [9] and Lazarova et al. [21]

Differentiated from *X*. *diffusum*, besides distant placement in LSU, ITS and *COI* mtDNA trees, by longer body (2497.7 (2124–2981) *vs* 1630 (1400–1800) μm), odontostyle and odontophore (113.5 (103–125) *vs* 85.5 (73–95) μm; and 68.3 (60–74) *vs* 49.7 (44–57) μm, respectively), more posteriorly located guiding ring (100 (91–112) *vs* 70.2 (60–77) μm) and greater body width at anus (35 (30–42) *vs* 22.8 (20–30) μm) [9].

Differentiated from *X*. *parabrevicolle*, the sister taxon in *COI* mtDNA tree by distant placement in LSU and ITS trees, longer odontophore (68.3 (60–74) *vs* 58.3 (54.5–61.0) μm), greater c′ value (0.9 (0.8–1.0) *vs* 0.7 (0.7–0.8)) and longer tail (31 (26–37) *vs* 26.6 (24.5–30) μm) [14].

Differentiated from *X*. *himalayense*, the tentative cryptic species of the new species, besides distant placement in both LSU and ITS trees, by lip region separated from the body by a constriction (*vs* continuous), conical tail, dorsally more convex (*vs* conoid, ventrally straight or slightly concave with rounded terminus) and wider body at anus (35 (30–42) *vs* 30.5 (28.9–32.4) [73].

Differentiated from *X*. *paramonovi* by a longer body (2497.7 (2124–2981) *vs* 2100 (2000–2300) μm), narrower lip region (12.5 (11–14) *vs* 14.6 (13.5–15) μm), longer odontophore (68.3 (60–74) *vs* 56.7 (53–60) μm), greater c value (81 (66.2–95.6) *vs* 60.5 (49.1–68)), smaller c′ value (0.9 (0.8–1.0) *vs* 1.1 (0.9–1.2)), greater body width at vulva (52.5 (41–65) *vs* 43.4 (39.0–47.2) μm) and shorter tail (31 (26–37) *vs* 36.1 (33–47) μm) [74].

Differentiated from *X*. *parataylori*, besides distant placement in LSU, ITS and *COI* mtDNA trees, by posteriorly located vulva (V = 54.7 (51.8–58.0) *vs* 49.7 (47–50)), longer odontostyle (113.5 (103–125) *vs* 91 (85–98) μm) and odontophore (68.3 (60–74) *vs* 58.6 (54–66) μm), posteriorly located guiding ring (100 (91–112) *vs* 78.5 (73–89) μm from anterior end) [21].

Differentiated from *X*. *taylori*, besides distant placement in LSU and *COI* mtDNA trees, by lip region separated from the rest body by a depression (*vs* constriction), longer body (2497.7 (2124–2981) *vs* 2300 (1800–2300) μm), odontostyle (113.5 (103–125) *vs* 95 (83.0–100.6) μm) and odontophore (68.3 (60–74) *vs* 58.8 (54.0–61.8) μm) and greater V value (54.7 (51.8–58.0) *vs* 50.4 (48–52)%), greater body width at anus (35 (30–42) *vs* 30.1 (25–34.1) μm) [75].

Differentiated from *X*. *incognitum*, besides distant placement in LSU, ITS and *COI* mtDNA trees, by longer body (2497.7 (2124–2981) *vs* 1900 (1700–2100) μm), greater a and c values (48 (39.2–61.7) *vs* 45 (41–49) and (81 (66.2–95.6) *vs* 62 (47–75), respectively), posteriorly located vulva (V = 54.7 (51.8–58.0) *vs* 51 (48–53%), longer odontostyle and odontophore (113.5 (103–125) *vs* 87 (82–93) μm, and (68.3 (60–74) *vs* 52 (46–56) μm, respectively), wider anal body region (35 (30–42) *vs* 28 (24–33) μm), and narrower tail tip (*vs* wider) [9].

Differentiated from *X*. *luci*, besides distant placement in LSU and ITS trees, by longer body (2497.7 (2124–2981) *vs* 1800 (1700–1900) μm), longer odontostyle and odontophore (113.5 (103–125) *vs* 95 (93–99) μm, and (68.3 (60–74) *vs* 50 (47–53) μm, respectively), posteriorly located guiding ring (100 (91–112) *vs* 76 (68–85) μm from anterior end), greater body width at anus (35 (30–42) *vs* 24 (20–30) μm), smaller c′ value (0.9 (0.8–1.0) *vs* 1.2 (1.0–1.4)) and posteriorly located vulva (V = 54.7 (51.8–58.0) *vs* 51 (49–52)%) [9].

**Iranian population of *X***. ***pachtaicum* from Amol**

(Fig 5)

**Fig 5:**
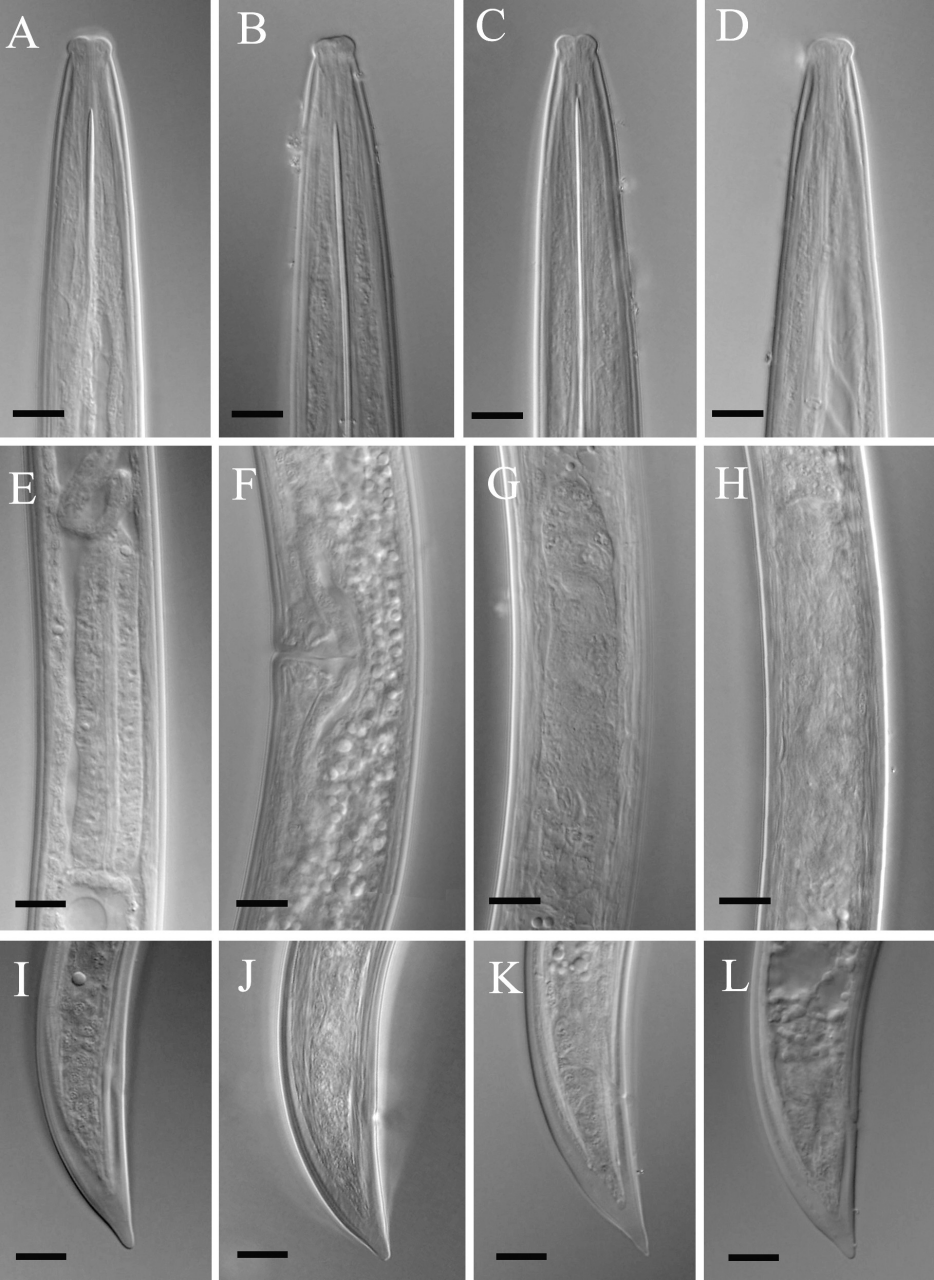
Light microphotographs of Iranian population of *Xiphinema pachtaicum* (Tulaganov, 1938) Kirjanova, 1951. (A-D) Anterior region. (E) Pharyngeal bulb. (F) Vulval region. (G and H) Endosymbiont bacteria inside the ovary. (I-L) Female tail. (All scale bars = 10 μm).

Two populations of the species were recovered from the rhizospheric soil samples of *Citrus* sp. and *Alnus* sp. trees, collected respectively in a garden and a forest close to the city of Amol in Mazandaran province, northern Iran (GPS coordinates 36°26′39″N, 52°19′8.8″E and 36°24′7.8″N, 52°18′13.05″E, respectively). The recovered populations were morphologically compared with some other populations of the species from different parts of the world. Compared with the Spanish populations reported by Gutiérrez-Gutiérrez et al. [13], Hungarian populations reported by Repasi et al. [76], and Yugoslavian populations reported by Barsi & Lamberti [20], no remarkable differences in the presently studied population were observed. Compared to the American populations described by Orlando et al. [10], differences in vulva location (V = 55.9–60.5% in Iranian populations *vs* 48–57%) were observed. As mentioned by Orlando et al. [10], American populations show differences with other populations of the species from other parts of the world for this index. Compared to the Iranian populations of the species reported by Fadaei et al. [77], only minor intraspecies variation was observed for body width at anus (17–24 *vs* 13.5–19 μm). Measurements are listed in Table 6.

**Table 6:** Morphometric data of *Xiphinema pachtaicum* (Tulaganov, 1938) Kirjanova, 1951 from Iran. All measurements in μm and in the format: mean ± s.d. (range).

Origin	Amol population from forest	Amol population from Garden
Characters	Female	Female
n	10	7
L	1865 ± 132	1940 ± 179
	(1607.5–2063.0)	(1706–2171)
a	58.8 ± 7.1	63.1 ± 6.3
	(44.3–68.8)	(57.4–72.8)
b	6.1 ± 0.4	6.5 ± 0.5
	(5.3–6.6)	(5.8–7.1)
c	62.7 ± 4.2	60.7 ± 5.7
	(55.2–68.8)	(51.7–65.7)
c'	1.6 ± 0.1	1.7 ± 0.1
	(1.5–1.8)	(1.6–1.8)
V	57.8 ± 2.3	58.2 ± 1.6
	(56.4–60.5)	(55.9–60.2)
Lip region height	3.7 ± 0.5	3.8 ± 0.4
	(3–4)	(3–4)
Lip region width	8.1 ± 0.3	8.0 ± 0.7
	(8–9)	(7–9)
Odontostyle length	85.7 ± 2.3	87.2 ± 2.0
	(82–91)	(85–89)
Odontophore length	49.7 ± 1.2	49.6 ± 1.8
	(47–51)	(47–52)
Total stylet	135.0 ± 2.8	137.0 ± 2.3
	(132–142)	(134–139)
Anterior end to guiding ring	76.1 ± 4.0	82.5 ± 2.1
	(70–83)	(80–85)
Pharynx	308.0 ± 14.6	298 ± 6
	(283–335)	(290–305)
Anterior genital branch	172 ± 22	199 ± 18
	(141–201)	(181–217)
Posterior genital branch	159 ± 13	194 ± 33
	(141–179)	(158–234)
Lip region-vulva distance	1077 ± 70	1129 ± 115
	(972–1180)	(991–1308)
Body width at mid-body	32.2 ± 5.0	30.8 ± 2.2
	(27–45)	(28–33)
- at anus	19.2 ± 1.9	18.4 ± 1.1
	(18–24)	(17–20)
- at base of pharynx	26.6 ± 1.2	27.0 ± 0.7
	(25–28)	(26–28)
Rectum	16 (n = 1)	−
Tail	30.0 ± 1.8	32 ± 2
	(28–33)	(30–35)
Hyaline region of tail	10.6 ± 1.7	10.8 ± 0.4
	(8–13)	(10–11)

#### Molecular phylogenetic relationships

**Molecular characterization of *Xiphinema primum* n. sp. and Iranian population of *X*.*pachtaicum***

**The basic local alignment search tool (BLAST) results using newly obtained sequences given in Table 3**

The BLAST search using partial LSU rDNA D2-D3 sequences of four isolates of the new species (MF372941, MF372942, MF372943, MF372947) revealed they have 99% identity with three accession numbers MH248814, MH248815 and AY601602 recorded in the GenBank as *Xiphinema* sp. Kra-BG, *Xiphinema* sp. Per-BG and *X*. *taylori*, respectively.

The BLAST search using ITS sequences of four isolates of the new species (MF372951, MF372952, MF372953, MF372954) revealed their identity with available sequences having a high coverage were never higher than 91%.

The BLAST search using *COI* mtDNA sequences of four isolates of the new species (MK202795, MK202796) revealed their identity with currently available sequences is at maximum 90%.

The BLAST search using partial sequences of LSU rDNA D2-D3 expansion segments of the Iranian isolates of *X*. *pachtaicum* (accession numbers MF372944, MF372945, MF372946) revealed they have 99–100% identity with sequences of other isolates of the species (KU250155, HM921389, KJ802888, HM921397, HM921365, HM921353, JQ990033, KP268968 and some other sequences).

The BLAST search using the 16S rDNA of the endosymbiont bacterium from three isolates of *X*. *pachtaicum*, revealed they have 99–100% identity with several sequences of Burkholderiacea bacterium (KT735078, KT735074, KT735068, KU899555, KU899554, KU899551, KT735076, KT735082 and some others) already isolated from the same species.

The BLAST search using the 16S rDNA of the endosymbiont bacterium from two isolates of *X*. *primum* n. sp. revealed they have at maximum, 95% identity with several sequences of *Candidatus* Xiphinematobacter spp. (KJ614453, KJ614452, KJ614450, AF217462, KJ614451, KT735101, KT735096).

The BLAST search using partial SSU rDNA of an isolate of *X*. *pachtaicum* sequenced during the present study (MF372940), revealed it has 99% identity with other sequences of the species (KU250139, KP407873, AM086682, MH484520) and an isolate of *X*. *penevi* Lazarova, Peneva & Kumari, 2016 [16] (KU250141) available in GenBank. No phylogenetic analyses were performed in the present study using this sequence.

#### The D2*-*D3 expansion segments of LSU rDNA phylogeny based on *Xiphinema primum* n. sp. and Iranian population of *X*. *pachtaicum* sequences

**Fig 6:**
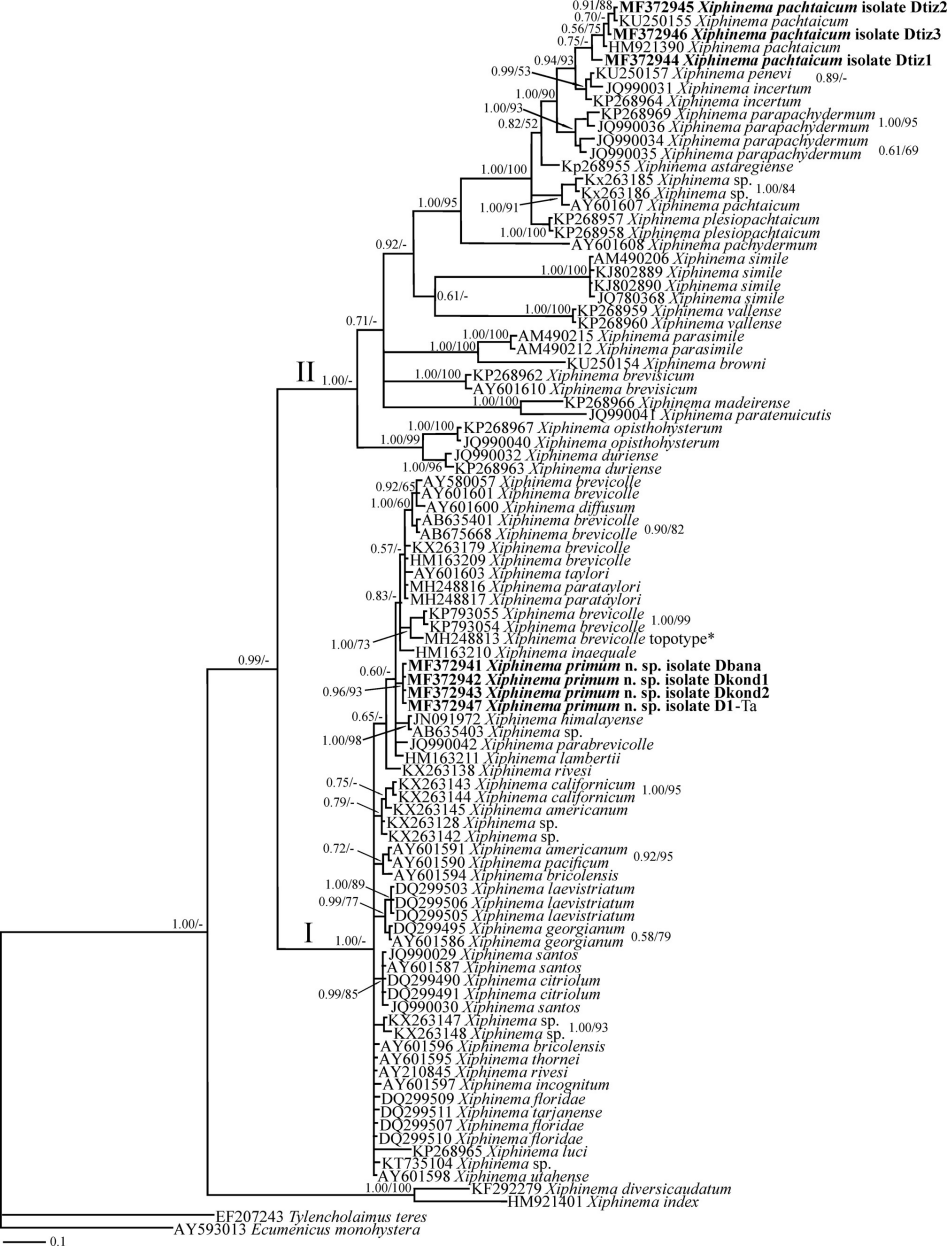
Bayesian 50% majority rule consensus tree inferred using LSU rDNA D2-D3 sequences of *X*. *primum* n. sp. and Iranian populations of *X*. *pachtaicum* under the GTR+G+I model. **Bayesian posterior probabilities and maximum likelihood bootstrap values are given for appropriate clades in the shape BPP/ML BS.** The newly generated sequences are in bold. According to Lazarova et al. [21], the accession number assigned to *X*. *brevicolle* marked by asterisk belongs to the topotype population of the species.

This dataset was composed of 727 characters of which 314 characters were variable. Fig 6 represents the Bayesian phylogenetic tree reconstructed using this dataset. The tree is divided to two major clades I and II, according to the nomenclature used in previous studies [10, 18]. In this tree, the new species appeared as an independent lineage in a clade containing *brevicolle*-complex spp. However, the relationships between most species in this clade were not resolved due to polytomy. The Iranian populations of *X*. *pachtaicum* formed a clade with two other selected isolates of the species (KU250155, HM921390). The five isolates of *X*. *pachtaicum* clade had almost identical LSU rDNA D2-D3 sequences (only one indel in overlapping region was observed), however, the length of their sequences differed, yielding intraspecies cladogenesis and varied branch lengths inside this species clade. The topotype population of *X*. *brevicolle* is marked by asterisk in this tree, corroborating its restricted distribution [21].

#### The ITS phylogeny based on *Xiphinema primum* n. sp. and Iranian population of *X*. *pachtaicum* sequences

**Fig 7:**
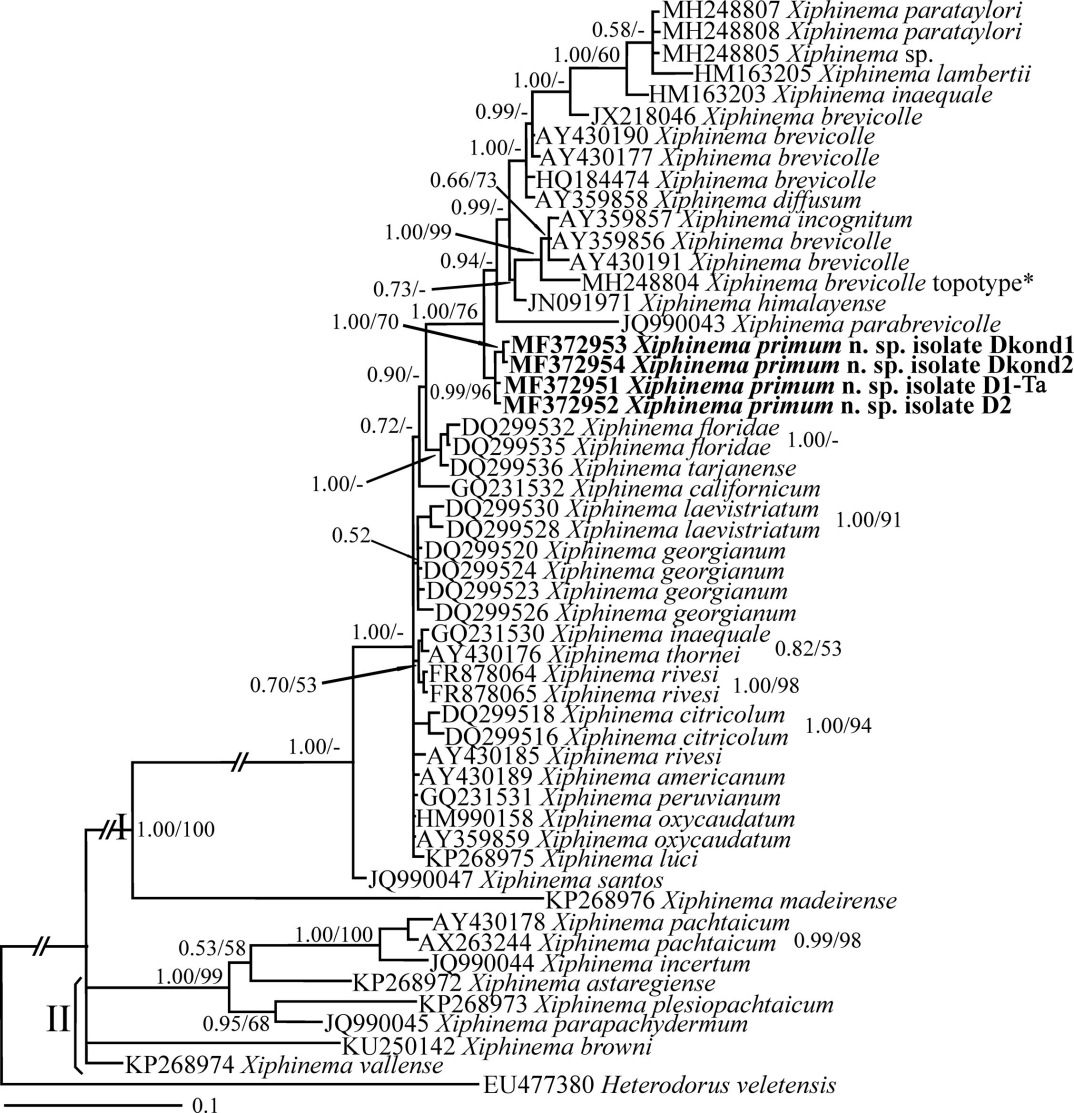
Bayesian 50% majority rule consensus tree inferred using ITS sequences of *X*. *primum* n. sp. under the GTR+G model. **Bayesian posterior probabilities and maximum likelihood bootstrap values are given for appropriate clades in the shape BPP/ML BS.** The newly generated sequences are in bold. According to Lazarova et al. [21], the accession number assigned to *X*. *brevicolle* marked by asterisk belongs to the topotype population of the species.

The ITS dataset was composed of 1077 characters of which 452 characters were variable. Fig 7 represents the Bayesian phylogenetic tree reconstructed using this dataset. In this tree, a similar pattern to that observed in LSU phylogeny was observed, i.e., the sequences in this tree were divided into two major clades I and II. However, the relationships between the clades inside group II were not resolved due to polytomy. The new species occupies a basal placement to the clade of the *X*. *brevicolle-*complex, mostly having similar morphology, and appeared as an independent lineage. Iranian isolates of *X*. *pachtaicum* are not included in this tree as attempts to get their ITS sequences failed. Again, the topotype population of *X*. *brevicolle* is marked by asterisk, corroborating its restricted distribution [21].

#### The *COI* mtDNA phylogeny based on *Xiphinema primum* n. sp. and Iranian population of *X*. *pachtaicum* sequences

**Fig 8:**
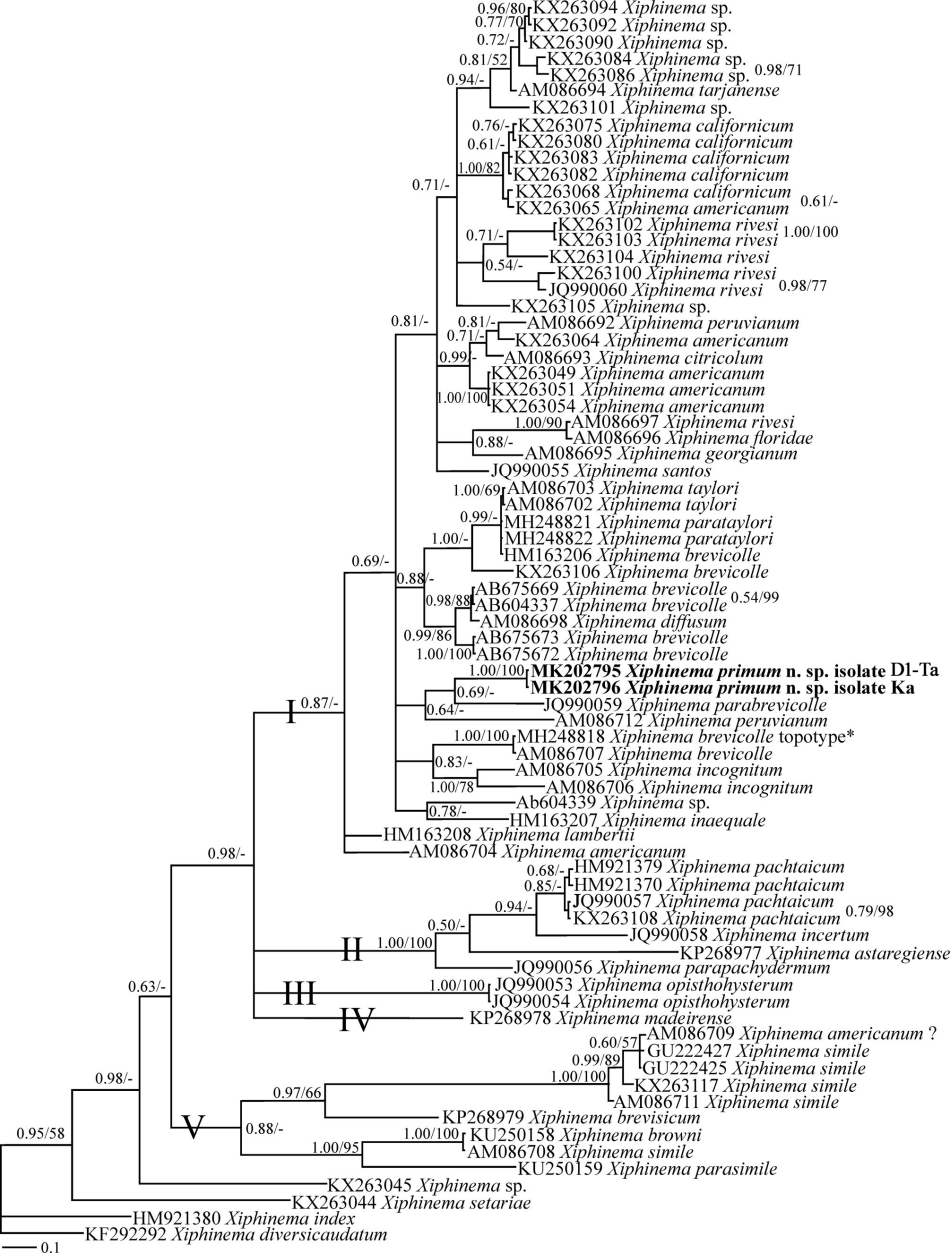
Bayesian 50% majority rule consensus tree inferred using *COI* mtDNA sequences of *X*. *primum* n. sp. under the GTR+G model. **Bayesian posterior probabilities and maximum likelihood bootstrap values are given for appropriate clades in the shape BPP/ML BS.** The newly generated sequences are in bold. According to Lazarova et al. [21], the accession number assigned to *X*. *brevicolle* marked by asterisk belongs to the topotype population of the species.

The *COI* dataset was composed of 348 characters of which 201 characters were variable. Fig 8 represents the Bayesian phylogenetic tree reconstructed using this dataset. In this tree, the relationship between the new species and other species and several members of the *X*. *brevicolle*-complex is not resolved due to polytomy, and it is sister to *X*. *parabrevicolle* and *X*. *peruvianum* Lamberti & Bleve-Zacheo, 1979 [9] with poor clade support. Similar to two previous phylogenies, the topotype population of *X*. *brevicolle* is marked by asterisk, corroborating its restricted distribution [21].

### Molecular characterization of endosymbiont bacterial species

#### *Ca*. Xiphinematobacter spp. phylogeny

**Fig 9:**
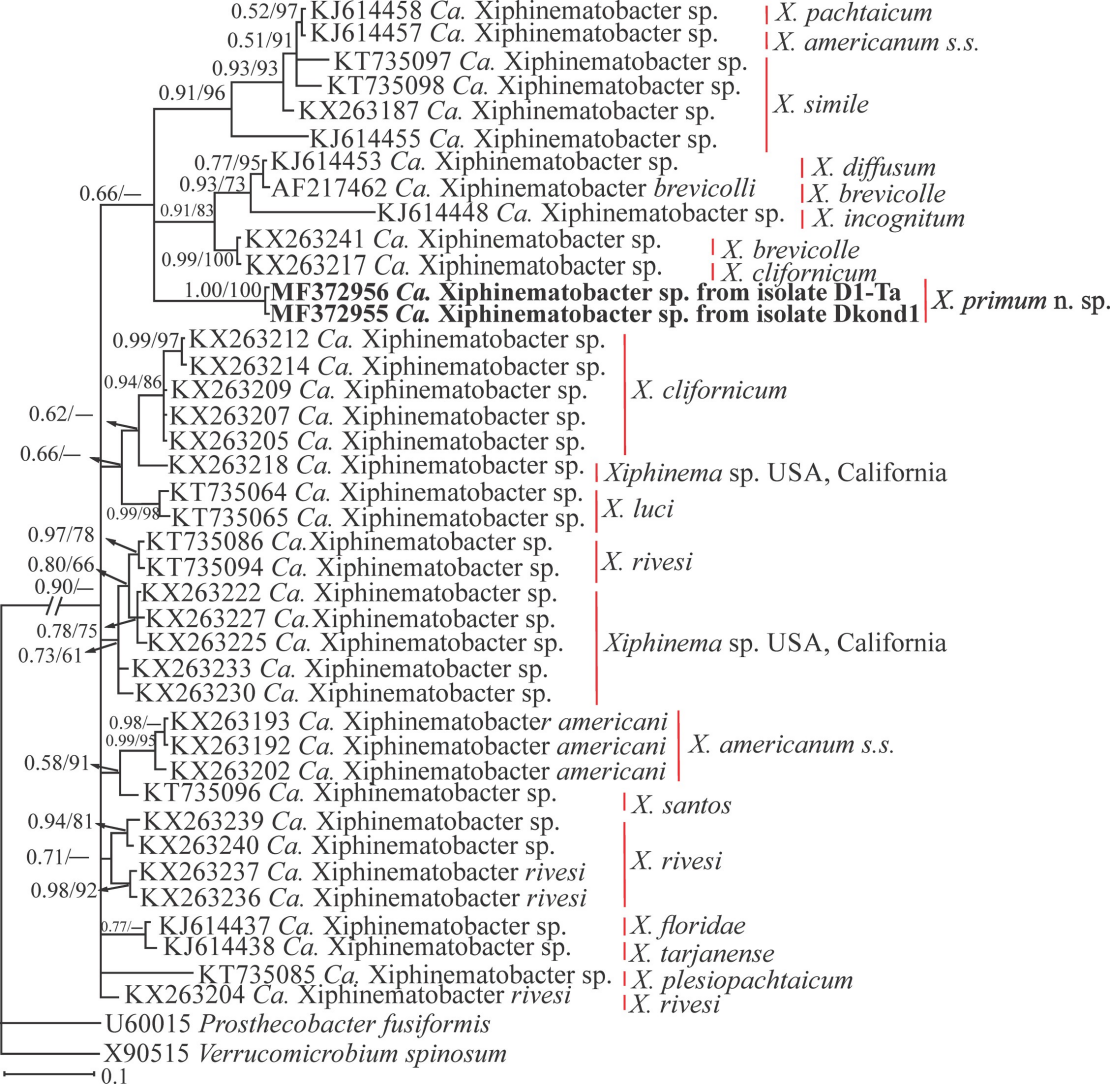
Bayesian 50% majority rule consensus tree inferred using 16S rDNA sequences of the endosymbiont bacteria, isolated from *Xiphinema primum* n. sp. under the GTR+G+I model. **Bayesian posterior probabilities and maximum likelihood bootstrap values are given for appropriate clades in the shape BPP/ML BS.** The newly generated sequences are in bold. The host nematodes are indicated in right.

The 16S rDNA dataset of *Ca*. Xiphinematobacter spp., including two newly amplified sequences of the bacterial 16S rDNA from the new species, included 526 total characters of which 158 characters were variable. Fig 9 represents the Bayesian phylogenetic tree reconstructed using this dataset. In this tree, the relation of the major clades was not resolved due to polytomy, and the newly generated sequences (MF372955, MF372956) were placed inside the clade including sequences of endosymbionts from *X*. *brevicolle*-complex (AF217462, KJ614448, KX263241, KJ614453). However, the clade received poor support (0.66/-) and different alignment, alignment post-editing, and reconstructing methods need to be applied to test any putative scenario for the relationship between these sequences.

#### The Burkholderiaceae spp. phylogeny

**Fig 10:**
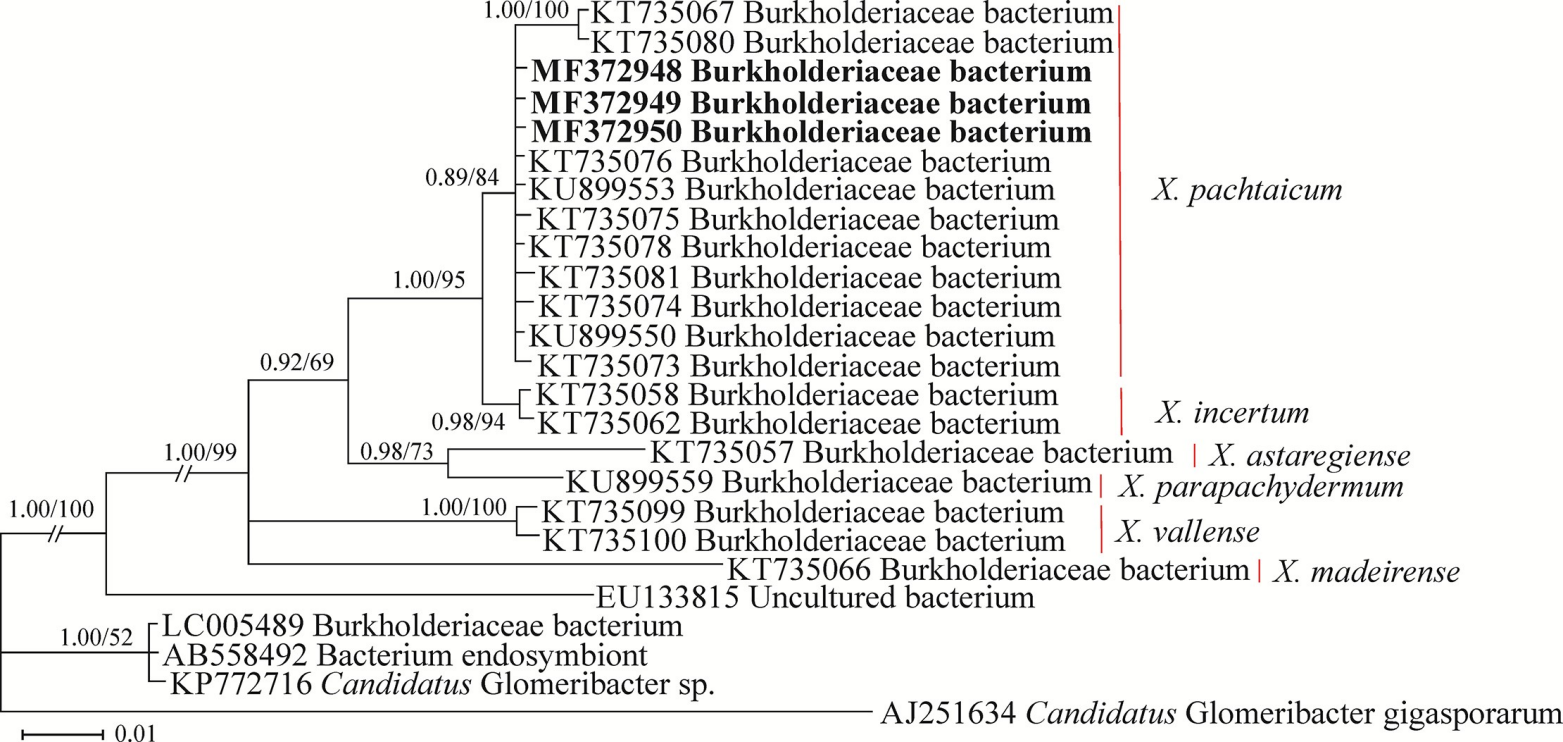
Bayesian 50% majority rule consensus tree inferred using 16S rDNA sequences of the endosymbiont bacteria, isolated from Iranian populations of *Xiphinema pachtaicum* under the GTR+G+I model. **Bayesian posterior probabilities and maximum likelihood bootstrap values are given for appropriate clades in the shape BPP/ML BS.** The newly generated sequences are in bold. The host nematodes are indicated in right.

The 16S rDNA dataset of Burkholderiaceae spp. including three newly generated sequences of endosymbionts from *X*. *pachtaicum* had 1228 characters of which 214 characters were variable. In this tree (Fig 10), the newly generated sequences occupied a placement inside the clade of several sequences of Burkholderiaceae from *X*. *pachtaicum*.

### Cophylogenetic analyses

**Fig 11:**
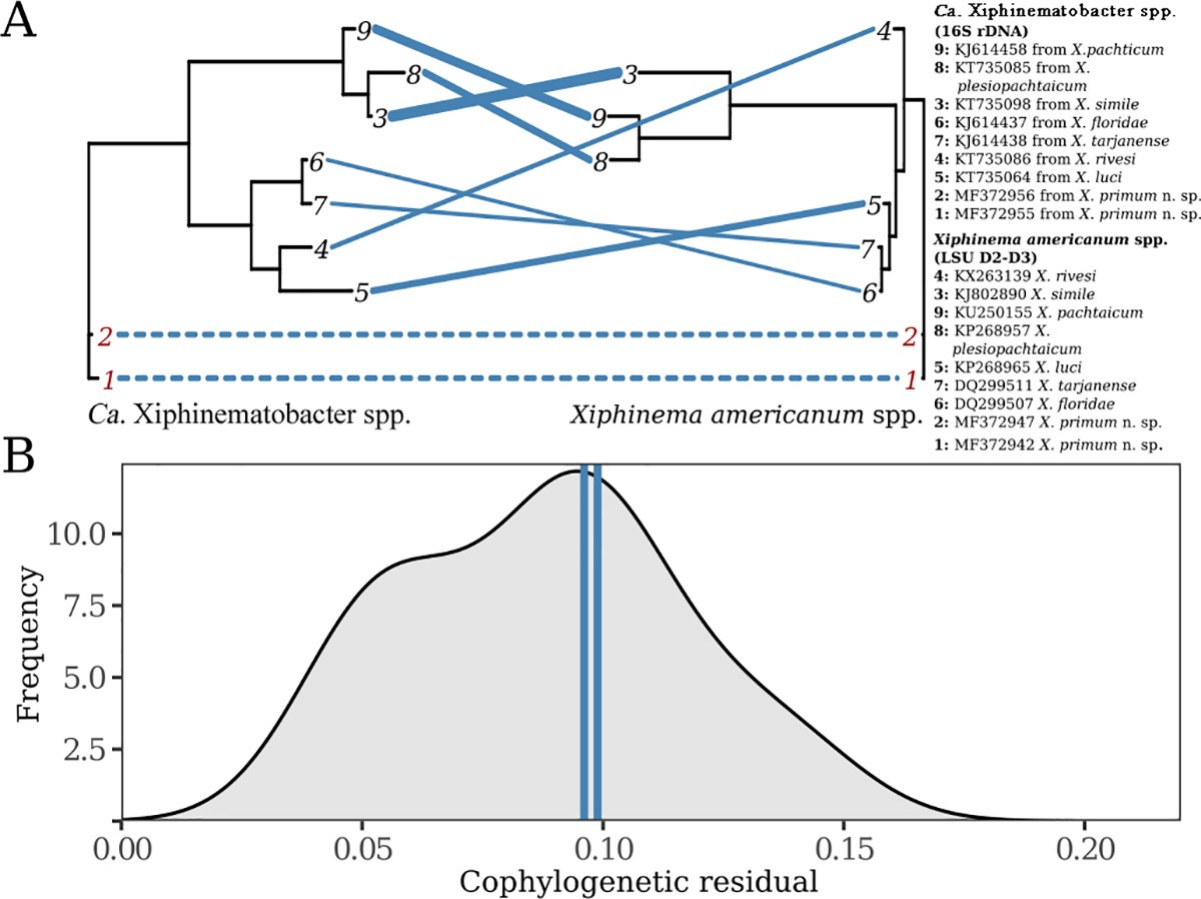
Cophylogenetic analysis of *Xiphinema americanum* spp. and their endosymbionts of *Ca*. Xiphinematobacter spp. using nematode LSU rDNA D2-D3 and bacteria 16S rDNA. (A) The phylogenies of endosymbiont bacteria alongside that of nematode host (*Xiphinema americanum* spp.). Blue lines connect host and endosymbiont and the thickness of each line represents the strength of cophylogenetic signal between host and endosymbiont. Thinner lines reflect stronger cophylogenetic signal (i.e. line thickness is the level of discordance between host and endosymbiont). Dashed blue lines and red numbers distinguish the species described in this study from the others used in the analysis. (B) The cophylogenetic signal of individual host-endosymbiont associations. Cophylogenetic signal is displayed on the x-axis where smaller values represent stronger cophylogenetic signal. The density profile (grey) describes the cophylogenetic signal between the all pairs of species and the two pairs identified in this study are also represented by the blue vertical lines.

**Fig 12:**
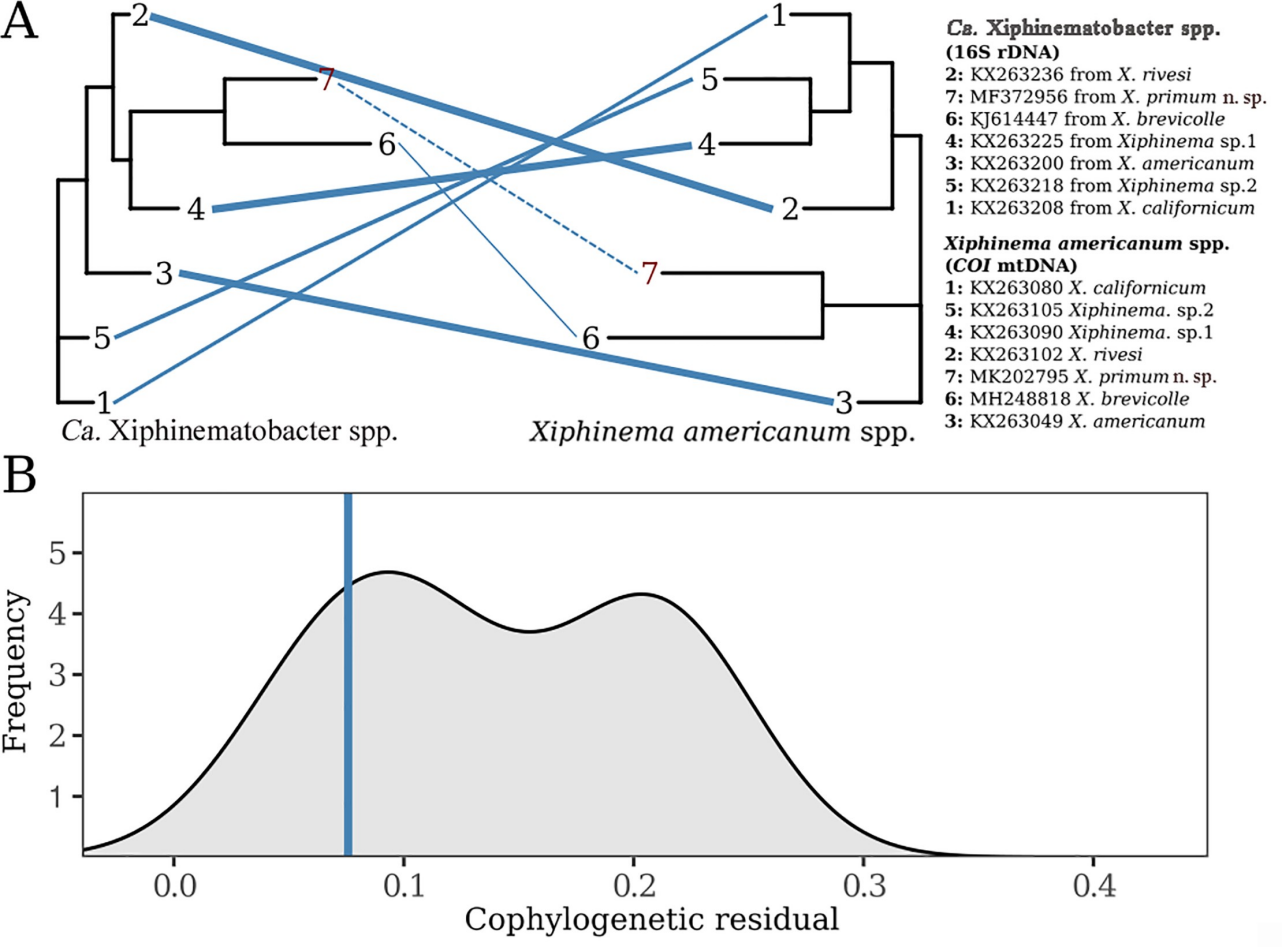
Cophylogenetic analysis of *Xiphinema americanum* spp. and their endosymbionts of *Ca*. Xiphinematobacter spp. using nematode *COI* mtDNA and bacteria 16S rDNA. (A) The phylogenies of endosymbiont bacteria alongside that of nematode host (*Xiphinema americanum* spp.) Blue lines connect host and endosymbiont and the thickness of each line represents the strength of cophylogenetic signal between host and endosymbiont. Thinner lines reflect stronger cophylogenetic signal (i.e. line thickness is the level of discordance between host and endosymbiont). Dashed blue lines and red numbers distinguish the species described in this study from the others used in the analysis. (B) The cophylogenetic signal of individual host-endosymbiont associations. Cophylogenetic signal is displayed on the x-axis where smaller values represent stronger cophylogenetic signal. The density profile (grey) describes the cophylogenetic signal between the all pairs of species and the pair identified in this study are also represented by the blue vertical line.

We examined the cophylogenetic patterns between selected sequences of *Xiphinema americanum*-group spp. and *Ca*. Xiphinematobacter spp. using both *COI* and LSU sequences for nematodes. In both cases, we observed strong, significant cophylogenetic signal between the two groups (LSU: ss = 0.076, n = 10,000, p < 0.01, Fig 11A; *COI*: ss = 0.168, n = 10,000, p < 0.01, Fig 12A). In Figs 11A and 12A, we plot the phylogenies of endsymbionts and hosts (LSU and *COI*, respectively) and blue lines connect host with endosymbiont where line thickness represents cophylogenetic signal strength (thinner lines represent smaller discrepancy between host and endosymbiont and therefore stronger cophylogenetic signal, dashed lines represent the species identified in this study). Only 0.5% and 0.1% of randomized host-endosymbiont associations, respectively, generated a stronger cophylogenetic signal than the observed associations. When comparing host-endosymbiont associations to each other, we saw that the endosymbiont-host pairs identified in this study display a medium level of cophylogenetic association (Figs 11B and 12B) with some associations (e.g. pairs 4, 6, 7 in Fig 11A and pair 6 in Fig 12A) showing stronger signal and others showing a weaker signal (e.g. pairs 3 & 9 in Fig 11A and pairs 1–5 in Fig 12A). In Figs 11B and 12B, we plot the distribution of cophylogenetic signal between pairs of hosts and endosymbionts and mark the species/populations described in this study with vertical lines that demonstrate their medium level of cophylogenetic signal relative to the rest of the group for the LSU marker and relatively strong signal with the *COI* marker. Overall, these results confirm the established pattern of strong cophylogenetic signal between these two groups, identify average levels of cophylogenetic signal between the host and endosymbiont introduced in this study, and provide another example of parallel cladogenesis between endosymbionts and their hosts.

## Discussion

The nematodes of the *Xiphinema americanum*-group are infamous for their difficulty in species delimitation using traditional criteria. The conserved morphology of even phylogenetically distant species or the close morphometric data ranges further complicate their identification. Using the mean values of the morphometric data as proposed by Orlando et al. [10] and applied in this study (see Differential and Diagnosis), could help primary species comparisons.

In this study, a new species in the *Xiphinema americanum-*group was described from Iran, as the first Iranian representative of the group, using an integrative approach. It belongs to *X*. *brevicolle*-complex, mainly characterized by a bluntly conoid tail, and the lip region not separated from the body by a sharp constriction. The species *X*. *himalayense* was regarded as its tentative cryptic species that besides morphological traits, and especially the tail characters, could be separated from it based on molecular data.

The molecular phylogenetic studies of the group were performed in some previous studies [10, 21, 34]. Using LSU rDNA, He et al. [12] found that the species of this group are divided into two subclades: the *X*. *americanum* and the *X*. *brevicolle* subgroups [12]. That latter was recently considered as “*X*. *brevicolle* species complex” [10]. The former phylogenetic studies based upon LSU rDNA D2-D3 and ITS markers, usually yielded congruent phylogenies [14], dividing the species into two main subclades I and II [10, 14, present study]. Two aforementioned studies, the present study, and the most recent study by Lazarova et al. [21] show that the *COI* mtDNA marker has acceptable interspecific variation to be used as an alternative approach that is helpful in overcoming the low divergence of LSU rDNA D2-D3 [10]. *X*. *primum* n. sp. was however separated from its morphologically close species and its cryptic species by both LSU and ITS markers in the corresponding phylogenetic trees.

The cryptic nature of some species and the misidentification of some species in GenBank remains an issue. The observed intraspecific divergence of D2-D3 marker in *X*. *brevicolle* s. l. [10, 14] documents the cryptic nature of nematodes of this group well. Sequencing of the topotype population of *X*. *brevicolle* s. str. by Lazarova et al. [21] revealed several populations are indeed misidentified and are erroneously assigned to this species. As a result, any observed paraphyletic or polyphyletic status of a given species, should be checked by the correct identification of that species first, and more preferably, topotype individuals of the studied species should be included in sequencing experiments whenever possible.

The recovered Iranian populations of *X*. *pachtaicum* and the new species were inspected for the presence of endosymbiont bacteria inside their ovaries. The rod-shaped endosymbiont bacteria were observed inside the ovaries of both recovered species under light microscopy (Fig 5G and 5H). The amplification of 16S rDNA and sequencing of the amplified fragments, revealed that the endosymbiont bacterium of the new species belongs to *Ca*. Xiphinematobacter sp., forming an independent lineage in the corresponding phylogenetic tree, and was not identical to other available sequences of this genus. The endosymbiont relation of *Ca*. Xiphinematobacter spp. with *X*. *americanum*-group members has recently been studied by several authors [34, 48, 10]. The endosymbiont of *X*. *pachtaicum* had an affinity with plant- and fungi-associated species of Burkholderiaceae, and was placed in the clade of species already isolated from *X*. *pachtaicum*. According to Palomares-Rius et al. [34], *X*. *pachtaicum* could harbor both endosymbionts; either a *Ca*. Xiphinematobacter sp. or a Burkholderiaceae sp.

Significant cophylogenetic signal has already been observed between nematodes of this group and both groups of the endosymbiont bacteria phylogenies, albeit with variation in signal strength [10, 34]. In this study, significant cophylogenetic signal was observed between both recovered nematode species and their endosymbionts phylogeny. The pair of species introduced in this study showed similar cophylogenetic signal to the rest of the group and do not disrupt the overall signal within the group. These results confirm the generally held expectation that host-endosymbiont evolution often occurs in parallel and indeed the evolution in the dependent group, parallels that of the host [66–68] as previously documented for *X*. *americanum*-group members and their endosymbionts [10, 34]. Indeed, the vertical transmission of endosymbionts via parthenogenesis means a significant cophylogenetic signal is expected.

## Conclusion

Hereby, we studied two Iranian populations of *Xiphinema pachtaicum* and described a new species, both of which belonged to the *X*. *americanum*-group, using an integrated approach. Significant cophylogeny between the newly generated sequences of nematode LSU rDNA D2-D3 and *COI* mtDNA sequences and the 16S rDNA sequences of the endosymbiont bacteria were also observed.
